# Complement C4b and factor B in astrocyte-derived exosomes as novel biomarkers for amnestic mild cognitive impairment

**DOI:** 10.3389/fnagi.2026.1727449

**Published:** 2026-09-30

**Authors:** Yu Li, Menglu Zhang, Chao Ying, Chao Han, Qimeng Li, Yanning Cai

**Affiliations:** 1 Department of Neurobiology, Xuanwu Hospital, Capital Medical University, Beijing, China; 2 Beijing Geriatric Medical Research Center, Beijing, China; 3 National Clinical Research Center for Geriatric Disorders, Beijing, China; 4 School of Rehabilitation and Exercise Health, Gannan Medical University, Ganzhou, Jiangxi, China; 5 National Clinical Research Center for Geriatric Disorders, Xuanwu Hospital, Capital Medical University, Beijing, China; 6 Department of Clinical Biobank and Central Laboratory, Xuanwu Hospital, Capital Medical University, Beijing, China

**Keywords:** amnestic mild cognitive impairment, *ApoE* ε4, astrocyte-derived exosomes, complement C4b, complement factor B

## Abstract

Neuroinflammation represents a fundamental pathological mechanism underlying amnestic mild cognitive impairment (aMCI), with dysregulated activation of the complement system playing a pivotal role. Astrocyte-derived exosomes (ADEs), which are capable of traversing the blood-brain barrier (BBB), offer a non-invasive means to investigate central nervous system pathology. This study explored the diagnostic potential of two principal complement components, C4b and factor B (FB), within plasma-derived ADEs for aMCI and analyzed their interaction with the *ApoE* ε4 allele. We enrolled 99 aMCI patients and 96 demographically matched normal controls (NC). C4b and FB levels in plasma-derived ADEs were quantified using ELISA, and all participants underwent *ApoE* genotyping. In the total cohort, levels of both C4b and FB in ADEs were significantly elevated in the aMCI group compared to NC. Diagnostic model validation demonstrated that adding C4b alone or in combination with FB to a baseline model significantly improved diagnostic performance, whereas adding FB alone provided no significant benefit. Notably, the *ApoE* ε4 genotype exerted a precise modulatory effect: among *ApoE* ε4 non-carriers, C4b levels were elevated in the aMCI group but did not yield significant diagnostic improvement; in contrast, among *ApoE* ε4 carriers, both C4b and FB were specifically upregulated, and only their combination achieved excellent diagnostic performance (AUC = 0.833; DeLong's test, *P* = 0.017), whereas neither marker alone was effective. Furthermore, C4b exhibited broader and more significant correlations with cognitive function compared to FB. This study substantiates that ADE-derived C4b serves as a reliable diagnostic biomarker for aMCI, whereas the diagnostic efficacy of FB is contingent upon *ApoE* ε4 allele status. Consequently, we propose a precision diagnostic strategy that involves the concurrent assessment of C4b and FB specifically in individuals carrying the *ApoE* ε4 allele, thereby presenting a novel approach for the early detection of aMCI.

## Introduction

Alzheimer's disease (AD) is a progressive neurodegenerative disorder and the leading cause of dementia worldwide. Its core clinical features include persistent cognitive decline, severe memory impairment, and loss of daily living abilities (Yu et al., 2021; Kamatham et al., 2024). With the accelerating trend of global population aging, the prevalence of AD is rising steadily, imposing a substantial burden on societies and healthcare systems (Honig et al., 2024). Amnestic mild cognitive impairment (aMCI) is recognized as a prodromal stage of AD, with an annual conversion rate to AD of 11.0%−16.5%, and is considered a critical window for early intervention between normal aging and clinical dementia (Ward et al., 2013; Cong et al., 2023; Abu-Rumeileh et al., 2024).

Current gold-standard biomarkers for AD predominantly depend on cerebrospinal fluid (CSF) analysis and positron emission tomography (PET). Although these methods exhibit high diagnostic accuracy, their routine clinical application is considerably constrained by their invasive nature, substantial cost, and limited accessibility (Xiao et al., 2021). Recently, blood-based biomarkers, such as the plasma Aβ42/40 ratio and phosphorylated Tau proteins, have emerged as promising alternatives and are progressively being integrated into the Amyloid, Tau, Neurodegeneration (ATN) diagnostic framework (Shi et al., 2024). Nevertheless, these biomarkers primarily address Aβ and Tau pathologies, thereby neglecting a critical aspect of AD pathogenesis, namely neuroinflammation. Consequently, the development of novel, minimally invasive, and reliable blood-based biomarkers that accurately reflect neuroinflammatory processes remains a pivotal scientific challenge for the early detection of aMCI.

In recent years, a substantial body of research has substantiated the pivotal role of neuroinflammatory responses, particularly the dysregulated activation of the complement system, in the pathogenesis of AD (Zorzetto et al., 2017). Considerable attention has been directed toward complement C4b, a common downstream component of both the classical and lectin pathways, and complement factor B (FB), the rate-limiting enzyme of the alternative pathway (Goetzl et al., 2018), both of which exhibit aberrant expression in the CSF of individuals with AD (Manral et al., 2012). The evidence implicating C4b in AD pathology is increasingly comprehensive: from increased gene copy number in peripheral blood (Zorzetto et al., 2017) to upregulated protein expression in affected brain regions (Canal-Garcia et al., 2025), multi-level evidence has collectively established its pathogenic potential. In contrast, FB exhibits dynamic expression characteristics associated with disease stage: although plasma FB levels in patients with established AD are generally lower than those in normal controls (NC) and individuals with MCI, baseline FB levels are significantly higher in MCI patients who subsequently progress to AD compared to those who do not progress (Morgan et al., 2019). This distinctive pattern suggests that FB may be involved in the multi-stage pathological progression of AD, with its expression dynamics reflecting the evolving neuroimmune status across different phases of the disease. In summary, the available evidence not only establishes the significant roles of C4b and FB in AD pathophysiology but also reveals their unique value as potential biomarkers.

The transformation of astrocytes from key supporting cells that maintain central nervous system homeostasis to a state of reactive gliosis has emerged as a core feature of the pathological process in AD and other neurodegenerative disorders (Kumar et al., 2023; Kim et al., 2024). As a significant source of complement factors within the brain, astrocytic dysfunction is closely linked to the pathogenesis of AD (Nogueras-Ortiz et al., 2020; Chen et al., 2021; Lin et al., 2023). Astrocyte-derived exosomes (ADEs), serving as key mediators in disease processes, can actively eliminate membrane-bound complement proteins through a protective clearance mechanism, or package newly synthesized and activation-induced covalently bound complement fragments via the exosomal biogenesis pathway (Pilzer et al., 2005; Colombo et al., 2014; Nogueras-Ortiz et al., 2020; Gu et al., 2023). These complement-carrying ADEs are capable of traversing the blood-brain barrier (BBB) to enter the peripheral circulation, thereby offering a unique avenue for non-invasive monitoring of central complement system activation (Kalluri and LeBleu, 2020; Xia et al., 2022). Multiple studies have confirmed that complement components within ADEs are closely associated with AD progression. Goetzl et al. (2018) found that levels of C4b and FB in ADEs were significantly elevated in patients with AD compared to controls, and that levels in patients with moderate AD were significantly higher than their own levels measured 5–12 years earlier when they were in the mild AD stage. In a prospective study, Winston et al. (2019) reported that MCI patients who converted to AD had higher levels of C4b and the Bb fragment (an activation product of FB) in their ADEs; similarly, Qi et al. (2023) found that serum ADEs from patients with type 1 diabetes mellitus accompanied by MCI exhibited elevated FB levels compared to those without MCI. These findings collectively suggest that astrocytes may deliver C4b and FB via the exosomal pathway, thereby participating in the pathological progression of AD. However, none of the aforementioned studies, whether comparing AD patients with NC, predicting MCI-to-AD conversion, or examining MCI in specific comorbidity contexts, have systematically evaluated C4b and FB in the aMCI stage. Therefore, the diagnostic value of C4b and FB in plasma ADEs for aMCI remains to be elucidated, and whether their diagnostic performance is modulated by key genetic factors warrants further investigation.

The polymorphism of the apolipoprotein E (*ApoE*) gene is recognized as the most significant genetic risk factor for AD, with the ε4 allele elevating the risk in a dose-dependent manner. This has positioned *ApoE* ε4 as a central focus in research on AD risk assessment and pathogenesis (Scheltens et al., 2021; Yu et al., 2021; Fortea et al., 2024). Studies have demonstrated that at the stage of aMCI, carriers of the *ApoE* ε4 allele not only exhibit a greater burden of AD pathology but also face a substantially increased risk of progression to clinical AD (Laczó et al., 2025). Nevertheless, genetic risk alone is inadequate for precise prediction of AD onset, highlighting the necessity of integrating genetic data with downstream pathophysiological processes for a comprehensive evaluation (Genin et al., 2011; Jin et al., 2021). Importantly, the pathogenic effects of *ApoE* ε4 are intricately associated with its regulation of neuroimmune homeostasis. Astrocytes, as the primary cellular source of *ApoE* in the brain, play a critical role in this regulation (Serrano-Pozo et al., 2021). Research indicates that the *ApoE* ε4 allele can induce astrocytes to adopt a pro-inflammatory phenotype through mechanisms such as dominant-negative effects and epigenetic regulation, potentially serving as a crucial link between genetic susceptibility and neuroinflammatory dysregulation (Arnaud et al., 2022). Based on these findings, we propose the central hypothesis that the *ApoE* ε4 genotype modulates the diagnostic value of C4b and FB for aMCI by specifically altering their expression levels within ADEs, likely through the induction of a pro-inflammatory state in astrocytes. Verifying this regulatory axis is crucial for elucidating the role of *ApoE* ε4 in the heterogeneous progression of aMCI and for developing precise predictive models based on exosomal complement biomarkers.

This study aims to investigate the diagnostic value of C4b and FB in ADEs for aMCI and to specifically evaluate the modulatory role of the *ApoE* ε4 genotype on their diagnostic performance. We anticipate that this research will provide novel, minimally invasive liquid biopsy biomarkers for the early diagnosis of aMCI and offer crucial theoretical foundation and practical basis for establishing *ApoE* genotyping-based precision diagnostic strategies.

## Materials and methods

### Participants

A total of 195 participants were ultimately enrolled in this study, comprising 99 aMCI and 96 NC. The study protocol was approved by the Ethics Committee of Xuanwu Hospital, Capital Medical University, and written informed consent was obtained from all participants prior to enrollment.

All participants underwent systematic baseline clinical assessments, which included the collection of demographic information, *ApoE* genotyping, and comprehensive neuropsychological evaluations across multiple cognitive domains. The cognitive assessment battery covered global cognition, memory, executive function, language, visuospatial skills, and neuropsychiatric symptoms. The specific neuropsychological scales and clinical assessment tools used are detailed in Supplementary Table 1.

The diagnosis of aMCI was established based on the Jak/Bondi actuarial neuropsychological criteria (Bondi et al., 2014). NC was defined as the absence of cognitive decline complaints from both the participant and an informant, with all core cognitive domains, including memory, executive function, language, attention, and visuospatial abilities, falling within the normal range of age and education adjusted norms on neuropsychological testing (Ma et al., 2024).

### *ApoE* genotyping

*ApoE* genotyping was performed to distinguish between ε2, ε3, and ε4 alleles by targeting two specific single-nucleotide polymorphisms (SNPs). Briefly, genomic DNA was extracted from peripheral blood leukocytes using the TIANamp Genomic DNA Kit (Tiangen Biotech, China) according to the manufacturer's instructions. The concentration and purity of the extracted DNA were quantified using a Nanodrop 2000 spectrophotometer (Thermo Fisher Scientific, USA). The target region of the *ApoE* gene was subsequently amplified by polymerase chain reaction (PCR) with specific primers (forward: 5′-ACGCGGGCACGGCTGTCCAAGG-3′; reverse: 5′-GGCGCTCGGGATGGCGCTGA-3′), as previously described by Wang et al. (2022). The amplification products were finally subjected to direct Sanger sequencing (Sangon Biotech, China) for definitive allele determination.

### Isolation and enrichment of ADEs

ADEs were isolated and enriched from plasma according to the following procedure. Briefly, 250 μL of thawed plasma was centrifuged at 12,000 × g for 15 min at 4 °C. The resulting supernatant was treated with 2 μL of thrombin (System Biosciences, USA) and incubated at 37 °C for 5 min, followed by another centrifugation step at 12,000 × g for 5 min at 4 °C. The collected supernatant was supplemented with phosphatase inhibitor (Thermo Fisher Scientific, USA) and protease inhibitor (Promega, USA), after which 63 μL of ExoQuick solution (System Biosciences, USA) was added. The mixture was incubated at 4 °C for 1 h to precipitate total exosomes. The resulting pellet was resuspended in 250 μL of buffer containing the aforementioned inhibitors.

To enrich for ADEs, the resuspended solution was incubated with 1.5 μg of mouse anti-human GLAST (ACSA-1) biotinylated antibody (Miltenyi Biotech, USA) for 2 h at 4 °C. Subsequently, 30 μL of streptavidin magnetic beads (MedChemExpress, USA) were added, and the mixture was incubated at room temperature for 1 h to form ADE-antibody-bead complexes. The complexes were isolated magnetically and washed three times with PBS containing 0.05% Tween-20. The final pellet was resuspended in 85 μL of IgG elution buffer and incubated at room temperature for 2 min. The eluate was collected by magnetic separation and neutralized with 15 μL of 1 M Tris-HCl (pH 7.5). The resulting eluate was mixed with 400 μL of Mammalian Protein Extraction Reagent (M-PER, Thermo Fisher Scientific, USA) containing protease and phosphatase inhibitors and lysed on ice for 30 min. The final 500 μL ADE lysate was stored at −80 °C for subsequent analysis.

### ADEs characterization

The ultrastructural morphology of isolated ADEs was visualized using transmission electron microscopy (TEM; FEI Tecnai Spirit, USA). Particle concentration and size distribution were determined by nanoparticle tracking analysis (NTA; NanoSight NS300, UK).

The identity of ADEs was further confirmed through Western blot analysis for specific marker proteins. ADE lysates were mixed with 5 × SDS loading buffer and denatured by heating at 100 °C for 10 min. Proteins were separated by SDS-PAGE and subsequently transferred onto PVDF membranes. The membranes were blocked with 5% skim milk for 1 h at room temperature, followed by incubation with the following primary antibodies at 4 °C overnight: rabbit anti-CD63 (1:1,000, System Biosciences, USA), rabbit anti-HSP70 (1:1,000, System Biosciences, USA), rabbit anti-calnexin (1:1,000, HUABIO, China), mouse anti-albumin (1:200, Bioss, China), mouse anti-glial fibrillary acidic protein (GFAP; 1:1,000, Abcam, UK), mouse anti-Alix (1:1,000, Cell Signaling Technology, USA), rabbit anti-CD171 (1:1,000, Abcam, UK), and rabbit anti-CD11b (1:1,000, Thermo Fisher Scientific, USA). After extensive washing with TBST buffer, the membranes were incubated with appropriate horseradish peroxidase (HRP)-conjugated secondary antibodies for 1 h at room temperature. Protein bands were finally visualized using a chemiluminescent substrate and imaged.

### Complement assay

The concentrations of C4b and FB in ADEs were quantified using commercial enzyme-linked immunosorbent assay (ELISA) kits according to the manufacturers' protocols (C4b: NOUVS, USA; FB: Thermo Fisher Scientific, USA). Briefly, ADE lysates were added to antibody-precoated microplates. After gentle washing, HRP-conjugated detection antibodies were applied. The colorimetric reaction was developed using tetramethylbenzidine (TMB) substrate, stopped with sulfuric acid solution, and the absorbance was measured at 450 nm using a microplate reader (Thermo Fisher Scientific, USA). Sample concentrations were determined based on standard curves.

To account for variations in exosome extraction efficiency, the measured levels of C4b and FB were normalized to the content of the exosomal marker CD81. The concentration of CD81 in ADE lysates was determined using a specific human CD81 ELISA kit (CUSABIO, China) following the same procedure described above. The normalized levels of complement factors are expressed as the ratio of C4b or FB concentration to CD81 concentration.

### Statistical analysis

All statistical analyses were performed using SPSS (version 27.0; IBM Corp., USA) and MedCalc (version 23.1.6; MedCalc Software Ltd., Belgium). Continuous variables were first tested for normality using the Shapiro-Wilk test. For demographic and clinical baseline characteristics, normally distributed continuous variables were presented as mean ± standard deviation (SD), non-normally distributed continuous variables as median (interquartile range, IQR), and categorical variables as number (percentage). Group comparisons between NC and aMCI participants were performed using the independent samples *t*-test for normally distributed continuous variables, the Mann—Whitney *U-*test for non-normally distributed continuous variables, and the chi-square test for categorical variables.

To evaluate group differences in complement factor levels within ADEs across the entire sample, linear regression models were constructed with adjustment for age, sex, years of education, and *ApoE* genotype. Furthermore, to analyze these differences within *ApoE* ε4 genotype subgroups (non-carriers vs. carriers), separate linear regression models were established with adjustment for age, sex, and years of education. The ability of each indicator to discriminate between the NC and aMCI groups was assessed using receiver operating characteristic (ROC) curve analysis, with the area under the curve (AUC) calculated. DeLong's test was subsequently employed to compare the statistical differences in AUC values between different ROC curves. Additionally, Spearman's correlation coefficient was used to analyze the relationships between complement factor levels and neuropsychological test scores. All tests were two-tailed, and *P* < 0.05 was considered statistically significant.

## Results

### Patient characteristics

In this study, a total of 195 participants were enrolled, consisting of 96 NC and 99 individuals diagnosed with aMCI. Baseline demographic and clinical characteristics for both cohorts are detailed in Table 1. Statistical analyses revealed no significant differences between the groups regarding age or sex distribution (both *P* > 0.05). However, the aMCI group showed significantly lower educational attainment (*P* < 0.001) and a higher prevalence of *ApoE* ε4 carriers (*P* = 0.038) compared with the NC group. Neuropsychological assessments demonstrated that the aMCI group scored significantly lower on the Mini-Mental State Examination (MMSE), Montreal Cognitive Assessment Basic (MoCA-B), Verbal Fluency Test (VFT), and Memory and Executive Screening (MES), while exhibiting significantly higher scores on the Clinical Dementia Rating (CDR) relative to the NC group (all *P* < 0.001). These five measures, integral to the clinical identification of aMCI, were therefore selected for subsequent correlation analyses with the biomarkers. Detailed results of the neuropsychological assessments are available in Supplementary Table 2. Supplementary Tables 3, 4 present the demographic and clinical characteristics of the subgroups of individuals who are non-carriers and carriers of the *ApoE* ε4 allele, respectively.

**Table 1:** Demographical and clinical characteristics.

Variables	NC (*n* = 96)	aMCI (*n* = 99)	*P*
Age, years	69.26 ± 7.98	70.28 ± 7.84	0.368
Female, *n* (%)	58 (60.42%)	61 (61.61%)	0.864
Education, years	14.00 (11.50, 16.00)	12.00 (9.00, 15.00)	< 0.001
*ApoE* ε4 carrier, *n* (%)	22 (22.92%)	35 (35.35%)	0.038
MMSE scores	29.00 (27.00, 30.00)	25.00 (23.00, 28.00)	< 0.001
MoCA-B scores	26.00 (24.00, 27.00)	21.00 (19.00, 23.00)	< 0.001
VFT scores	19.55 ± 4.56	14.53 ± 4.57	< 0.001
MES scores	91.00 (85.00, 96.00)	76.00 (64.00, 85.00)	< 0.001
CDR scores	0.00 (0.00, 0.00)	0.50 (0.50, 0.50)	< 0.001

Data are presented as mean ± SD, median (IQR), or *n* (%). For group comparisons, see Statistical analysis section. *P* < 0.05 was considered statistically significant. NC, normal controls; aMCI, amnestic mild cognitive impairment; *ApoE* ε4, Apolipoprotein E ε4; MMSE, mini-mental state examination; MoCA-B, Montreal cognitive assessment-basic scores; VFT, verbal fluency test; MES, memory and executive screening; CDR, clinical dementia rating.

### Characterization of ADEs in plasma

The morphology of the isolated ADEs was initially characterized through TEM, which identified typical cup-shaped structures with an approximate diameter of 100 nm in both NC and aMCI groups (Figure 1A). Subsequent analysis of their physical properties was conducted using NTA, revealing mean particle diameter of 109 ± 40.2 nm for the NC group and 126.6 ± 41.5 nm for the aMCI group (Figure 1B).

**Figure 1:**
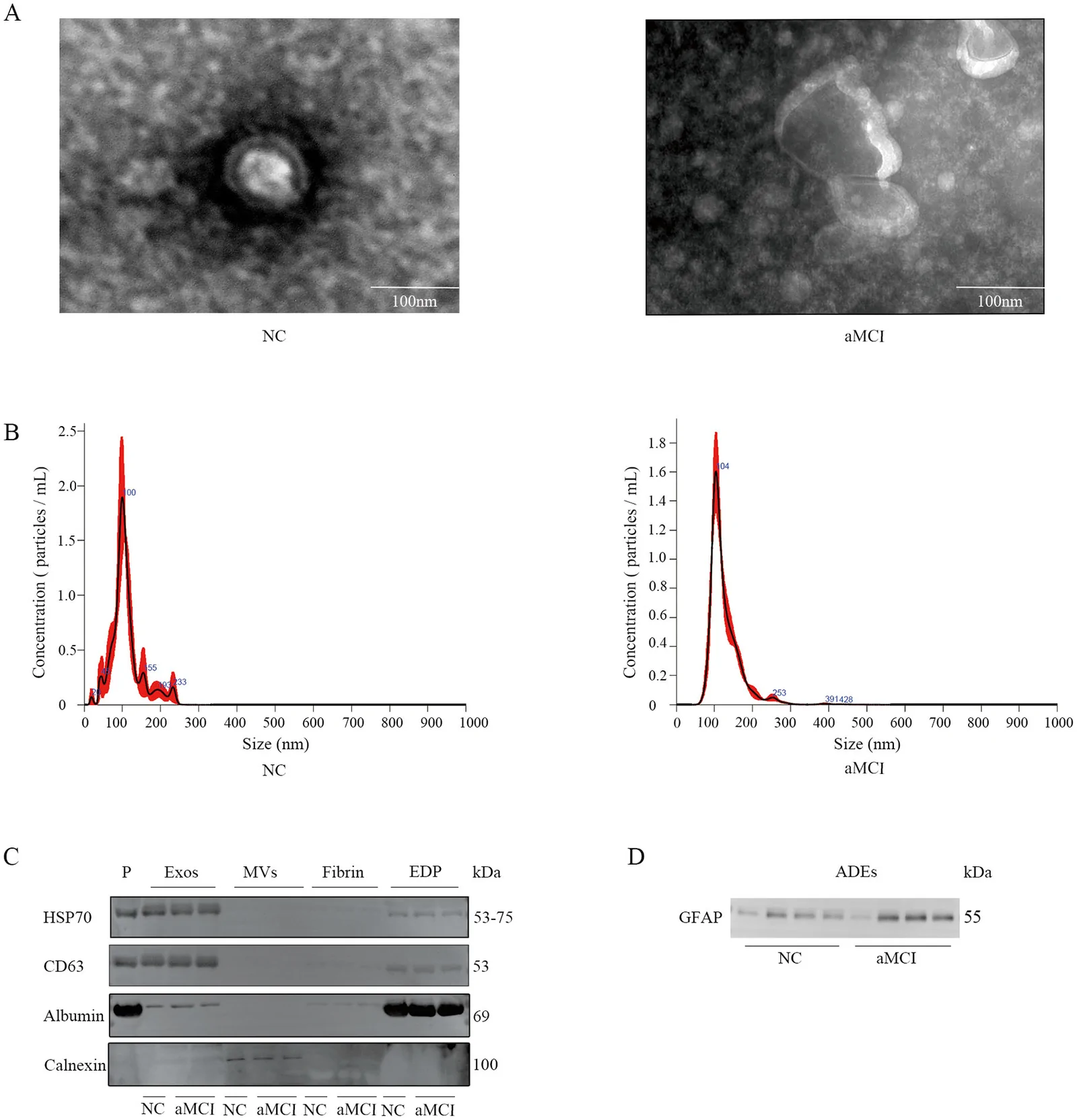
Characterization of ADEs isolated from plasma. **(A)** Representative TEM image of ADEs from an NC subject and an aMCI patient, showing the cup-shaped morphology of ADEs. Scale bar: 100 nm. **(B)** Representative NTA results of ADEs from an NC subject and an aMCI patient. The average particle diameter was found to be 109 ± 40.2 nm in the NC group, whereas it was 126.6 ± 41.5 nm in the aMCI group. **(C)** Representative Western blot analysis showing specific markers in sequential fractions obtained during the total exosome isolation procedure: plasma **(**P), microvesicle-enriched pellet after initial centrifugation (MVs), fibrin pellet after thrombin treatment (Fibrin), total exosomes precipitated by ExoQuick (Exos), and the final exosomes-depleted plasma (EDP). Results are presented for one representative NC subject and two representative aMCI patients. **(D)** Representative Western blot analysis from four representative NC subjects and four representative aMCI patients, confirming the enrichment of the astrocyte-specific marker GFAP in ADEs. ADEs, astrocyte-derived exosomes; GFAP, glial fibrillary acidic protein; NC, normal control; aMCI, amnestic mild cognitive impairment.

Next, protein markers were characterized through Western blot analysis. Figure 1C systematically illustrates the results of the marker protein analysis across various fractions obtained during the total exosome isolation process. These fractions comprise the initial plasma, microvesicle-enriched pellets following initial centrifugation (MVs), fibrin pellets formed after thrombin treatment, total exosomes precipitated using ExoQuick, and the final exosome-depleted plasma supernatant. The results showed that the exosomal marker proteins HSP70 and CD63 exhibited high expression levels in both plasma and the total exosome fraction. In contrast, the commonly co-isolated protein Albumin was abundant in plasma and exosomes-depleted plasma but was minimally detected in the total exosome fraction. The negative marker Calnexin was exclusively identified in the MVs fraction and was absent from the initial plasma, total exosome fraction, or exosomes-depleted plasma. This phenomenon can be principally ascribed to two factors: the enhancement of the signal is achieved through the enrichment of MVs via centrifugation, and the presence of high-abundance proteins in plasma has the potential to obscure low-abundance targets. These findings corroborate that our exosome preparations were devoid of contamination from larger vesicles or cellular components.

Additionally, Western blot analysis confirmed that the astrocyte-specific marker glial fibrillary acidic protein (GFAP) was highly expressed in immunoaffinity-enriched ADEs (Figure 1D). Full, uncropped images of all Western blots are provided in Supplementary Figures 1A–E. To systematically evaluate the purity of the obtained ADEs, we examined specific markers of exosomes derived from different cell types. The results demonstrated that while ADE samples highly expressed the exosomal marker protein Alix and the astrocyte marker GFAP, they were negative for the neuron-derived exosome marker CD171 and the microglia-derived exosome marker CD11b (Supplementary Figure 1F), confirming that this isolation strategy effectively avoided cross-contamination with exosomes from other cellular origins.

### Expression, diagnostic performance, and clinical correlations of complement components in the total cohort.

We first quantified the levels of C4b and FB in ADEs across the total cohort. In a linear regression model adjusted for age, sex, years of education, and *ApoE* genotype, the aMCI group exhibited significantly higher levels of both C4b (*P* < 0.001; Figure 2A) and FB (*P* = 0.041; Figure 2B) in ADEs compared to the NC group. Results from the unadjusted analysis are provided in Supplementary Figure 2.

**Figure 2:**
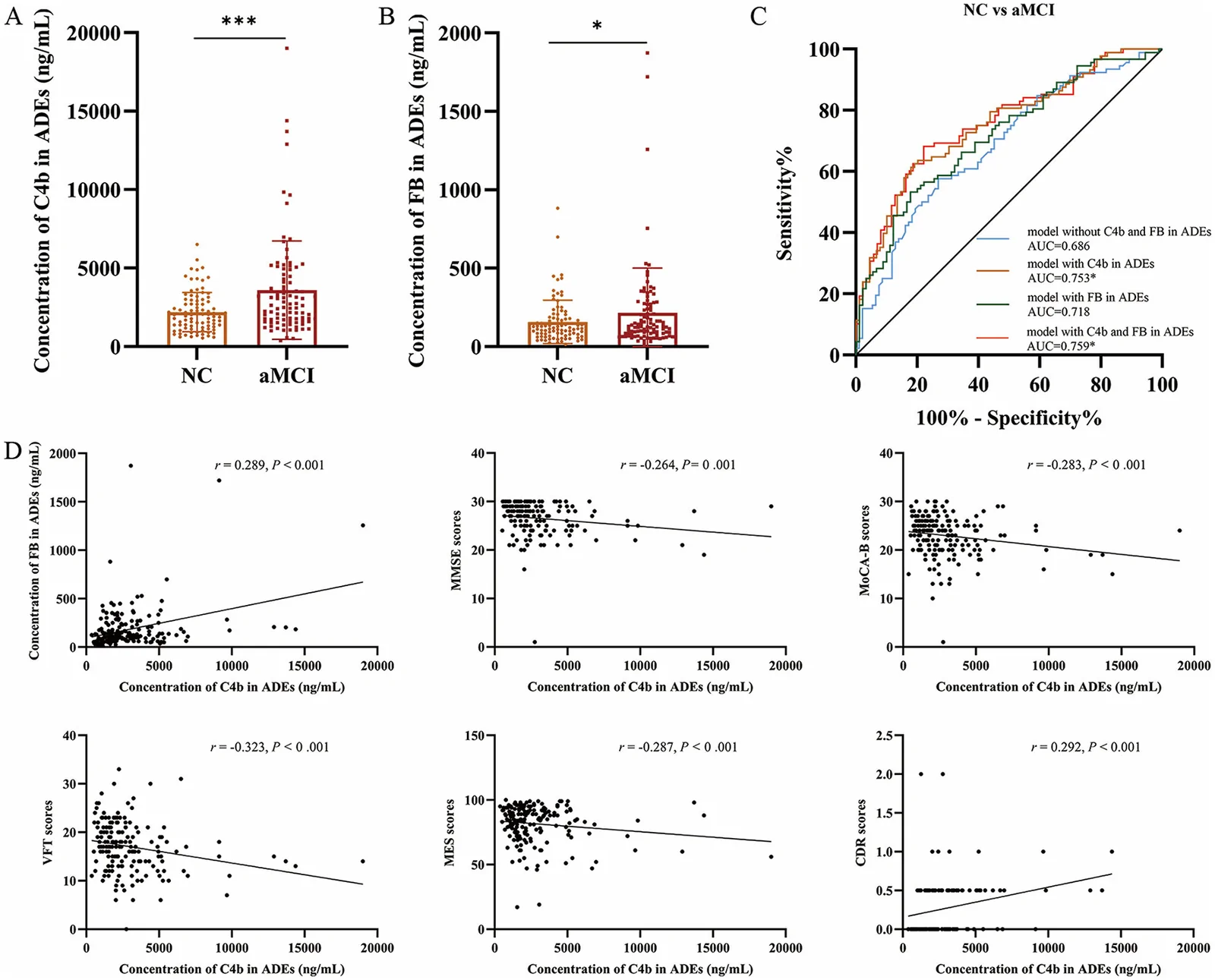
Expression, diagnostic performance, and clinical correlations of complement components in ADEs from the entire cohort. **(A, B)** Comparison of C4b **(A)** and FB **(B)** levels in ADEs between NC and aMCI. These analyses were conducted using linear regression models, with adjustments made for age, sex, education, and *ApoE* ε4 genotype. The sample sizes were NC, *n* = 96, and aMCI, *n* = 99. *P < 0.05; ***P < 0.001. **(C)** ROC curve analysis of different logistic regression models for discriminating aMCI from NC. The baseline model incorporated age, sex, education, and *ApoE* ε4 genotype, while extended models were developed by adding C4b alone, FB alone, or both C4b and FB to the baseline model. The ROC curves and the AUC values are depicted for each model. DeLong's test was employed to compare the AUCs of the extended models with that of the baseline model, *P < 0.05. **(D)** Correlation analyses of C4b with FB and neuropsychological scores in ADEs. Spearman's rank correlation coefficient was used given the non-normal distribution of the data. *P* < 0.05 considered statistically significant. ROC, receiver operating characteristic; AUC, area under the curve; NC, normal control; aMCI, amnestic mild cognitive impairment; ADEs, astrocyte-derived exosomes.

To assess the added diagnostic value of C4b and FB in discriminating aMCI, we constructed a baseline logistic regression model incorporating clinical covariates (age, sex, years of education, and *ApoE* genotype). Three extended models were subsequently developed by separately adding C4b, separately adding FB, and jointly adding both C4b and FB. ROC analysis revealed that the baseline model achieved an AUC of 0.686 (95% *CI*: 0.609–0.762, *P* < 0.001). The addition of C4b alone increased the AUC to 0.753 (95% *CI*: 0.682–0.824, *P* < 0.001), while adding FB alone resulted in an AUC of 0.718 (95% *CI*: 0.644–0.791, *P* < 0.001). The joint inclusion of both C4b and FB further improved the AUC to 0.759 (95% CI: 0.688–0.830, *P* < 0.001). DeLong's test indicated that, compared to the baseline model, the inclusion of C4b alone (*P* = 0.039) or the combination of C4b and FB (*P* = 0.028) resulted in statistically significant improvements in diagnostic performance (Figure 2C).

We conducted Spearman's correlation analysis to investigate the relationships between C4b and FB levels in ADEs and their associations with neuropsychological assessment scores. The analysis demonstrated a significant positive correlation between C4b and FB levels (*r* = 0.289, *P* < 0.001). At the neuropsychological level, increased C4b levels were significantly correlated with poorer performance across several cognitive domains, as indicated by lower scores in global cognitive function (MMSE: *r* = −0.264, *P* = 0.001; MoCA-B: *r* = −0.283, *P* < 0.001), verbal fluency (VFT: *r* = −0.323, *P* < 0.001), and memory and executive function (MES: *r* = −0.287, *P* < 0.001), as well as higher disease severity (CDR: *r* = 0.292, *P* < 0.001) (Figure 2D). Conversely, FB levels did not exhibit significant correlations with any of the neuropsychological measures. Comprehensive correlation results for both C4b and FB with all neuropsychological scores are detailed in Supplementary Table 5.

### Expression, diagnostic performance, and clinical correlations of complement components in *ApoE* ε4 non-carriers.

To elucidate the modulatory role of *ApoE* ε4 genotype on complement factors, we analyzed the expression profiles of C4b and FB in the *ApoE* ε4 non-carrier subgroup. After adjusting for sex, age, and years of education, the aMCI group showed significantly elevated C4b levels in ADEs compared to the NC group (*P* = 0.004; Figure 3A), whereas FB levels remained comparable between the two groups (*P* > 0.05; Figure 3B). Results from the unadjusted analysis are presented in Supplementary Figure 3.

**Figure 3:**
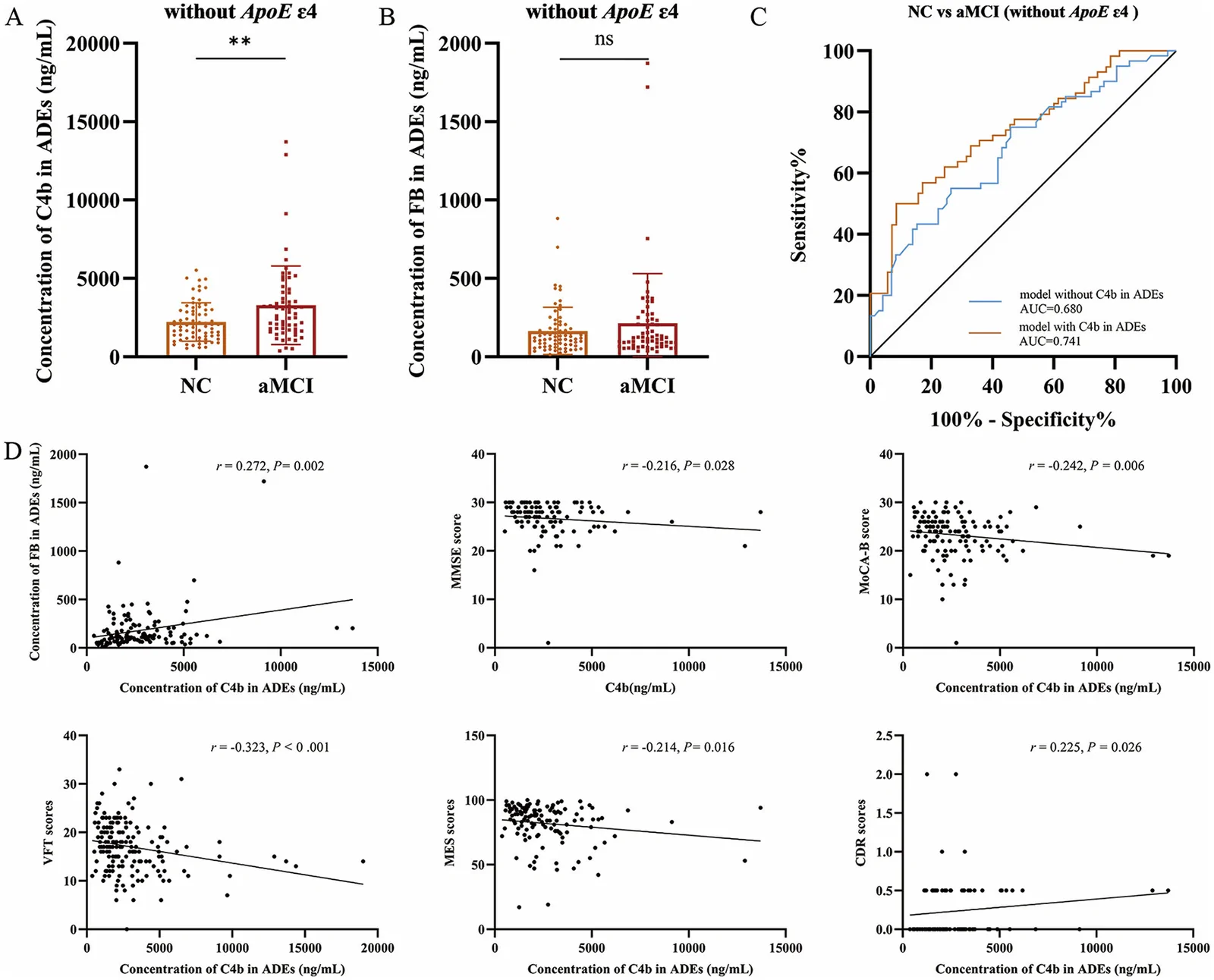
Expression, diagnostic performance, and clinical correlations of complement components in ADEs from *ApoE* ε4 non-carriers. **(A, B)** Comparison of C4b **(A)** and FB **(B)** levels in ADEs between NC and aMCI among *ApoE* ε4 non-carriers. These analyses were conducted using linear regression models, with adjustments made for age, sex, and education. The sample sizes were NC, *n* = 74, and aMCI, *n* = 64. **P < 0.01; ns, not significant. **(C)** ROC curve analysis of logistic regression models for discriminating aMCI from NC in *ApoE* ε4 non-carriers. The baseline model incorporated age, sex, and education, while the extended model added C4b to the baseline variables. The ROC curves and the AUC values are depicted for each model. DeLong's test indicated no significant difference in AUC between the two models (*P* > 0.05). **(D)** Correlation analyses of C4b with FB and neuropsychological scores in ADEs from *ApoE* ε4 non-carriers. Spearman's rank correlation coefficient was used given the non-normal distribution of the data. *P* < 0.05 considered statistically significant. ROC, receiver operating characteristic; AUC, area under the curve; NC, normal control; aMCI, amnestic mild cognitive impairment; ADEs, astrocyte-derived exosomes. *ApoE* ε4, Apolipoprotein E ε4.

ROC analysis indicated that the baseline model, which included only demographic variables (sex, age, and years of education), exhibited limited ability to discriminate between aMCI and NC (AUC = 0.680, 95% *CI*: 0.589 −0.772, *P* < 0.001). The inclusion of C4b into the baseline model enhanced its diagnostic accuracy (AUC = 0.741, 95% *CI*: 0.655 – 0.827, *P* < 0.001). Nonetheless, DeLong's test revealed that the increase in AUC was not statistically significant (*P* > 0.05; Figure 3C). Additionally, the incorporation of FB, either alone or in conjunction with C4b, did not significantly enhance diagnostic performance (DeLong's test, *P* > 0.05; Supplementary Figure 4). These findings suggest that C4b and FB do not contribute additional diagnostic value in this cohort.

To investigate the interrelationship between C4b and FB and their respective associations with neuropsychological performance in *ApoE* ε4 non-carriers, we performed correlation analyses within this subgroup. The results demonstrated a significant positive correlation between C4b and FB levels (*r* = 0.272, *P* = 0.002). Notably, even after adjusting for *ApoE* ε4, C4b levels showed significant correlations with multiple cognitive domains, including global cognition (MMSE: *r* = −0.216, *P* = 0.028; MoCA-B: *r* = −0.242, *P* = 0.006), language function (VFT: *r* = −0.323, *P* < 0.001), memory and executive function (MES: *r* = −0.214, *P* = 0.016), and disease severity (CDR: *r* = 0.225, *P* = 0.026) (Figure 3D). These findings indicate that in *ApoE* ε4 non-carriers, C4b demonstrates independent and broad associations with cognitive impairment in aMCI, while FB shows no significant clinical correlations. Complete data are available in Supplementary Table 6.

### Expression, diagnostic performance, and clinical correlations of complement components in *ApoE* ε4 carriers.

In the subgroup of *ApoE* ε4 carriers, after adjusting for covariates, the levels of C4b (*P* = 0.004) and FB (*P* = 0.038) in ADEs were significantly elevated in the aMCI group compared to the NC group (Figures 4A, B). Results from the unadjusted analysis are presented in Supplementary Figure 5.

**Figure 4:**
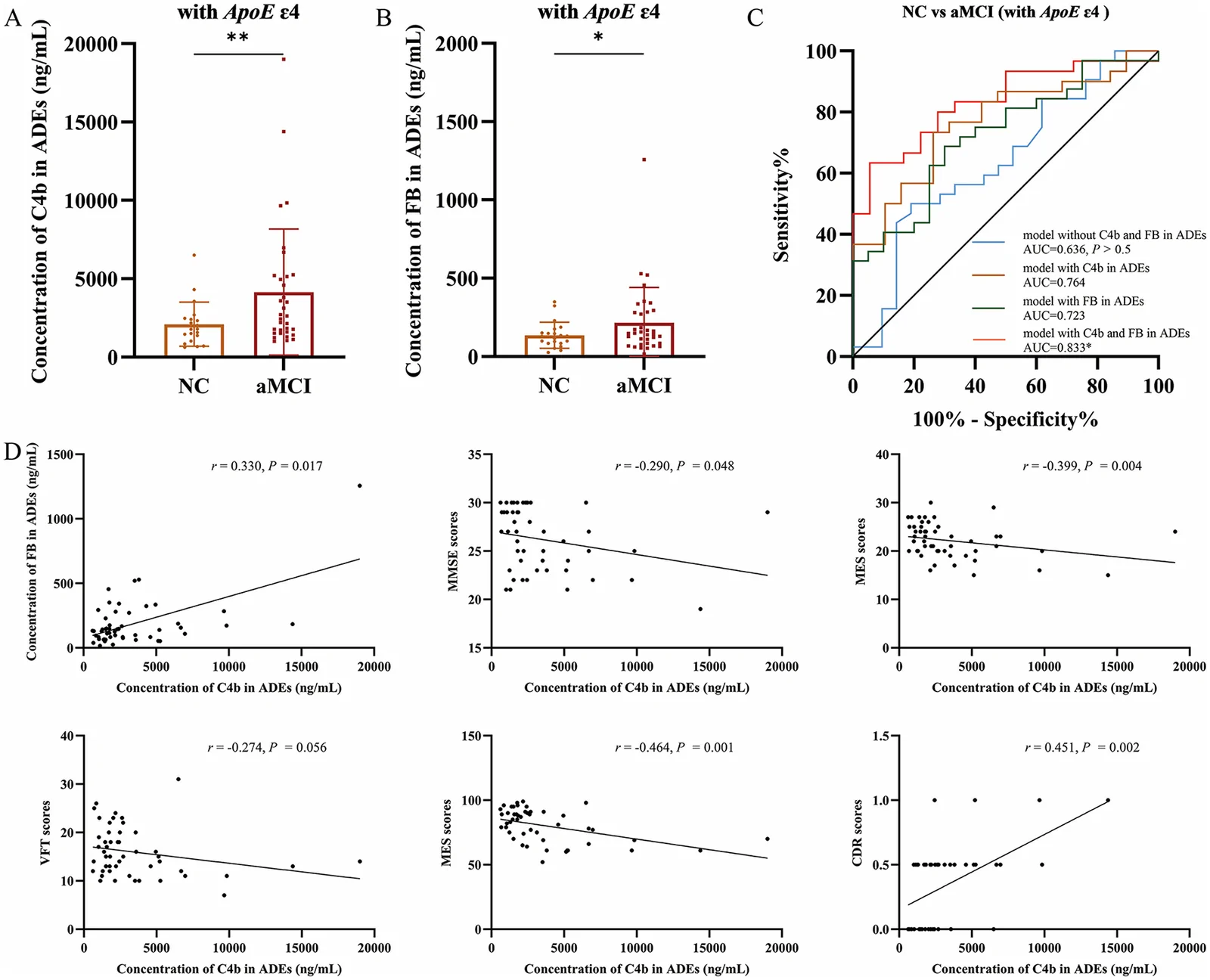
Expression, diagnostic performance, and clinical correlations of complement components in ADEs from *ApoE* ε4 carriers. **(A, B)** Comparison of C4b **(A)** and FB **(B)** levels in ADEs between NC and aMCI among *ApoE* ε4 carriers. These analyses were conducted using linear regression models, with adjustments made for age, sex, and education. The sample sizes were NC, *n* = 22, and aMCI, *n* = 35. *P < 0.05; **P < 0.01. **(C)** ROC curve analysis of different logistic regression models for discriminating aMCI from NC in *ApoE* ε4 carriers. The baseline model incorporated age, sex, and education, while extended models were developed by adding C4b alone, FB alone, or both C4b and FB to the baseline model. The ROC curves and the AUC values are depicted for each model. DeLong's test was used to compare the AUCs of the extended models with that of the baseline model, *P < 0.05. **(D)** Correlation analyses of C4b with FB and neuropsychological scores in ADEs from *ApoE* ε4 carriers. Spearman's rank correlation coefficient was used given the non-normal distribution of the data. *P* < 0.05 considered statistically significant. ROC, receiver operating characteristic; AUC, area under the curve; NC, normal control; aMCI, amnestic mild cognitive impairment; ADEs, astrocyte-derived exosomes. *ApoE* ε4, Apolipoprotein E ε4.

ROC analysis indicated that the baseline model, which included only demographic covariates, was inadequate in effectively distinguishing between aMCI and NC (*P* > 0.05). The integration of either C4b or FB into the baseline model enhanced its discriminatory capacity (C4b model: AUC = 0.764, 95% *CI*: 0.634–0.899, *P* = 0.002; FB model: AUC = 0.723, 95% *CI*: 0.585–0.862, *P* = 0.007). Nonetheless, DeLong's test demonstrated that the inclusion of C4b or FB individually did not yield statistically significant improvements in diagnostic performance compared to the baseline model (both *P* > 0.05). Conversely, when both C4b and FB were incorporated into the model, the combined diagnostic performance was optimized (AUC = 0.833, 95% *CI*: 0.720–0.947, *P* < 0.001). DeLong's test confirmed that the enhancement in AUC for the combined model relative to the baseline model was statistically significant (*P* = 0.017; Figure 4C).

In the *ApoE* ε4 carrier subgroup, C4b and FB maintained a significant positive correlation (*r* = 0.330, *P* = 0.017). C4b levels showed negative correlations with global cognitive function (MMSE: *r* = −0.290, *P* = 0.048; MoCA-B: *r* = −0.399, *P* = 0.004) and memory and executive function (MES: *r* = −0.464, *P* = 0.001), while demonstrating a positive correlation with disease severity (CDR: *r* = 0.451, *P* = 0.002). Notably, in contrast to the *ApoE* ε4 non-carrier subgroup, C4b showed no significant association with language function (VFT: *P* > 0.05) in ε4 carriers (Figure 4D), whereas FB exhibited a significant positive correlation with disease severity (CDR: *r* = 0.374, *P* = 0.010; Supplementary Table 7). The findings indicate that the *ApoE* ε4 genotype may influence the range of clinical phenotypes associated with the pathological role of C4b. Furthermore, the relationship between FB and disease severity appears to be closely linked to the *ApoE* ε4 status, highlighting the intricate interactions between the complement pathways and genetic background. Comprehensive results are presented in Supplementary Table 7.

## Discussion

Aberrant activation of the complement system plays a crucial role in the pathogenesis of neurodegenerative disorders, such as AD (Dalakas et al., 2020; Krance et al., 2021; Zelek and Morgan, 2022; Zelek et al., 2025). Research indicates that complement components may be released into the extracellular microenvironment via ADEs, thereby contributing to the cascade and dissemination processes associated with neuroinflammation (Upadhya et al., 2020). Importantly, in contrast to the systemic, nonspecific inflammatory state indicated by peripheral blood complement levels (Krance et al., 2021; Burgelman et al., 2022; Gutierrez et al., 2024), complement components transported by plasma ADEs, owing to their capacity to traverse the BBB, provide a more precise and direct reflection of the pathophysiological alterations occurring within the central nervous system (Jia et al., 2020; Upadhya et al., 2020).

This study conducted a systematic evaluation of the diagnostic potential of C4b, a crucial component of both the classical and lectin complement pathways, and FB, a regulatory factor of the alternative pathway, within ADEs for the detection of aMCI. Additionally, the study explored the influence of the *ApoE* ε4 genotype on the expression levels and diagnostic efficacy of these components. The principal findings are summarized as follows: (1) C4b was identified as a robust biomarker, consistently showing elevated levels across all cohorts and demonstrating significant correlations with multi-domain cognitive impairment; (2) The diagnostic utility of FB was found to be strictly context-dependent, with a notable synergistic effect observed exclusively in the *ApoE* ε4 carrier subgroup; (3) Within this specific genetic context, the combination of C4b and FB achieved high diagnostic discrimination for aMCI (AUC = 0.833). Overall, these findings indicate that complement components derived from ADEs have significant potential as minimally invasive blood-based biomarkers for aMCI and support the viability of a precision diagnostic approach stratified by *ApoE* ε4 genotype.

As a pivotal effector molecule in both the classical and lectin complement pathways, C4b is integral to immune recognition and the modulation of inflammatory responses. Its activation is initiated by the proteolytic cleavage of C4 by C1s within the C1 complex, generating C4a and C4b. C4b then covalently binds to target surfaces via an exposed thioester bond and associates with C2 to form the C3 convertase (C4b2a). This complex subsequently cleaves C3, thereby amplifying the complement cascade and enhancing immune opsonization and inflammatory regulation (Tenner, 2020; Nimmo et al., 2024). In this study, we demonstrated for the first time that C4b levels are significantly elevated in peripheral blood ADEs from patients with aMCI, consistent with previous findings in AD (Goetzl et al., 2018; Winston et al., 2019), indicating that aberrant complement activation occurs in the early stages of the AD spectrum. Further analysis revealed that C4b levels were significantly correlated with multiple cognitive domain scores, suggesting a clear dose-response relationship between the extent of complement activation and impairment in specific cognitive domains. Notably, this association was specifically modulated by the *ApoE* ε4 genotype. In the carrier subgroup, the correlation between C4b and VFT disappeared, suggesting that this genetic risk factor may reshape the target range of complement-mediated neurotoxicity. The underlying mechanisms may involve aberrant synaptic pruning and neuronal dysfunction mediated by the C1q-C4-C3 signaling axis (Lian et al., 2015; Wu et al., 2019; Veremeyko et al., 2023).

As a fundamental regulatory protein in the alternative complement pathway, FB is pivotal in complement activation (Tenner, 2020). Its activation constitutes a crucial step in the formation of the C3/C5 convertase (C3bBb), thereby facilitating the subsequent amplification of the alternative pathway cascade (Kavanagh et al., 2025). In this study, we observed that the expression pattern and clinical significance of FB are intricately modulated by the *ApoE* ε4 genotype. Specifically, FB levels were markedly elevated and demonstrated a positive correlation with disease severity, as assessed by the CDR score, exclusively in *ApoE* ε4 carriers. This distinct expression pattern is consistent with the complex dynamic changes in FB previously documented across the AD spectrum (Morgan et al., 2019), reinforcing the notion that FB is not an independent biomarker. Its pathophysiological relevance must be interpreted in the context of specific genetic backgrounds and within the broader framework of pathway activation.

The primary finding of our study lies in revealing a hierarchical regulatory pattern, wherein the complement biomarkers C4b and FB are intricately modulated by *ApoE* ε4 in the context of aMCI diagnosis. Within the overall cohort, C4b demonstrated robust baseline diagnostic value, with either its individual addition or its combination with FB significantly enhancing diagnostic performance (AUC = 0.753 and 0.759, respectively). Nevertheless, an analysis stratified by *ApoE* ε4 status revealed a more intricate pattern: neither biomarker alone significantly enhanced diagnostic accuracy in either ε4 carriers or non-carriers. However, in ε4 carriers, the combination of C4b and FB exhibited a significant synergistic effect, achieving optimal diagnostic performance (AUC = 0.833; DeLong's test, *P* = 0.017). This pattern indicates that the substantial synergistic role of FB is specific to the *ApoE* ε4 carrier subgroup rather than being universally applicable. Recent studies suggest that the *ApoE* ε4 allele plays a pivotal role in driving the transition of astrocytes to a pro-inflammatory phenotype. Specifically, *ApoE* ε4 downregulates Transgelin 3 (TAGLN3) expression, which in turn alleviates the inhibition of NF-κB, thereby activating inflammatory pathways (Arnaud et al., 2022). This activation may directly enhance the synthesis of complement components C4b and FB. Additionally, *ApoE* ε4 disrupts astrocytic lipid metabolism and lysosomal function (Windham and Cohen, 2023; Blumenfeld et al., 2024), potentially hindering the clearance of complement-coated debris and redirecting excess complement proteins into exosomal release pathways, thereby intensifying peripheral complement signals. These alterations in astrocytic homeostasis induced by *ApoE* ε4 may synergistically amplify C4b and FB signals specifically in *ApoE* ε4 carriers, which accounts for the superior diagnostic efficacy of the combined detection strategy in this subgroup. Consequently, the *ApoE* ε4 allele may serve as a pathological “amplifier” (Martens et al., 2022; Raulin et al., 2022; Parhizkar and Holtzman, 2022), offering a genetic rationale for the enhanced diagnostic performance of C4b and FB. These findings underscore the critical importance of integrating genetic background and pathway interactions in biomarker development, thereby offering novel insights for the precision diagnosis and mechanistic understanding of aMCI. Importantly, the synergistic interaction between C4b and FB, representing the classical/lectin and alternative complement pathways, respectively, may be specific to certain diseases and is not consistently observed across all inflammatory conditions.

Overall, our findings are consistent with those of Goetzl et al. (2018) and further extend the association of complement activation to the earlier aMCI stage, suggesting that complement activation may constitute an early driving event in the AD pathological cascade. The complement profiling utilizing peripheral blood ADEs presents distinct advantages: compared to lumbar puncture-dependent CSF analysis and costly PET imaging, this method demonstrates superior accessibility, reproducibility, and patient acceptability, making it well-suited for large-scale screening and long-term dynamic monitoring (Su et al., 2021). In this study, we propose for the first time an innovative *ApoE* ε4-stratified precision diagnostic strategy for aMCI. Specifically, for ε4 non-carriers, C4b alone enables cost-effective universal screening; conversely, for ε4 carriers, the combined assessment of C4b and FB ensures high-accuracy identification. This stratified approach suggests that a single, low-cost *ApoE* genotyping test can effectively determine the most appropriate detection pathway for aMCI screening, thereby providing a viable solution to balance “precision” with “universality” at the public health level. This strategy not only enhances the diagnostic workflow but also holds significant potential as a critical recruitment tool for future precision prevention and intervention studies targeting genetically defined subpopulations. Nevertheless, the clinical translation of this stratified strategy faces certain limitations. Despite the significant differences in complement levels in ADEs observed between groups and their correlations with cognitive scores, their capacity to discriminate at the individual level remains limited. This suggests that the current findings possess greater biological significance than immediate diagnostic utility. Consequently, this stratified combined detection strategy constitutes merely an initial exploration toward precision diagnosis. Future endeavors should focus on integrating multimodal prediction models and standardized detection technologies to further enhance its clinical applicability.

Furthermore, this study has several limitations. Primarily, the cross-sectional nature of the study impedes the ability to ascertain whether fluctuations in C4b and FB can serve as predictors for the progression from aMCI to AD, highlighting the need for future validation through prospective cohort studies. Additionally, the sample size for subgroup analyses was relatively small, necessitating the validation and refinement of the proposed precision diagnostic model in larger, independent cohorts. Third, the precise molecular mechanisms by which *ApoE* ε4 influences complement factors require further investigation through cellular and animal studies. Fourth, the complex and time-consuming method used to extract ADEs in this study limits its clinical use. To make it a practical biomarker tool, future studies need to simplify and standardize the isolation process.

In conclusion, this study substantiates that ADE-derived C4b functions as a reliable minimally invasive blood-based biomarker for aMCI, and elucidates for the first time that the diagnostic utility of FB is intricately modulated by the *ApoE* ε4 genotype. These findings offer novel potential methodologies for the early detection and risk stratification of aMCI and establish a theoretical framework for the development of precision intervention strategies tailored to populations with varying *ApoE* ε4 genetic profiles.

## Data Availability

The datasets generated and analyzed during this study will be provided by the corresponding authors upon reasonable request.
