# Supplementary material for: Complement C4b and factor B in astrocyte-derived exosomes as novel biomarkers for amnestic mild cognitive impairment
Source: Front Aging Neurosci. 2026 Sep 30;18:1727449. doi: 10.3389/fnagi.2026.1727449 (PMC13661567; doi:10.3389/fnagi.2026.1727449)
Supplement: Supplementary file 1 [file Data_Sheet_1.pdf]

Supplementary Table 1 Comprehensive list of neuropsychological and clinical assessments

Assessment Domain	Assessment Tool (Abbreviation)	Primary Purpose and Cognitive Domains Measured
**Global Cognition**	Mini-Mental State Examination (MMSE)	A brief screening tool for global cognitive impairment, assessing orientation, memory, attention, calculation, and language.
	Montreal Cognitive Assessment Basic (MoCA-B)	A sensitive tool for screening mild cognitive impairment, assessing visuospatial/executive function, naming, memory, attention, language, abstraction, and orientation.
**Language**	Boston Naming Test (BNT)	Evaluates visual confrontation naming ability and word retrieval.
	Verbal Fluency Test (VFT)	Assesses semantic memory retrieval and verbal fluency.
**Executive Function & Attention**	Shape Trail Test-A (STT-A)	Measures visual search, attention, and processing speed.
	Shape Trail Test-B (STT-B)	Assesses task switching, cognitive flexibility, and complex executive function.
**Memory & Executive Screening**	Memory and Executive Screening (MES)	A composite tool for screening impairments in both memory and executive functions.
**Mood**	Hamilton Depression Scale (HAMD)	Assesses the severity of depressive symptoms.
	Hamilton Anxiety Scale (HAMA)	Assesses the severity of anxiety symptoms.
**Disease Severity & Staging**	Global Deterioration Scale (GDS)	Provides a global measure of the severity of cognitive decline.
	Clinical Dementia Rating (CDR)	A semi-structured interview to stage the severity of dementia by evaluating cognitive and functional performance.
**Daily Function**	Functional Activities Questionnaire (FAQ)	Assesses instrumental activities of daily living; a key criterion for diagnosing MCI and dementia.

This table provides a comprehensive summary of the assessment tools employed to evaluate multiple cognitive and clinical domains, including global cognition, memory, executive function, language, visuospatial skills, mood, daily functioning, and disease severity. The table presents the specific assessment tool (abbreviation), its primary purpose, and the cognitive domains measured.

Supplementary Table 2 Comprehensive neuropsychological assessment of the total study cohort

**Variables**	**NC (n = 96)**	**aMCI (n = 99)**	***P***
MMSE scores	29.00 (27.00, 30.00)	25.00 (23.00,28.00)	< 0.001
MoCA-B scores	26.00 (24.00, 27.00)	21.00 (19.00, 23.00)	< 0.001
BNT scores	26.00 (24.00, 28.00)	22.00 (19.00, 25.00)	< 0.001
VFT scores	19.55 ± 4.56	14.53 ± 4.57	< 0.001
STT-A (seconds)	54.00 (44.00, 64.50)	81.00 (59.50, 108.00)	< 0.001
STT-B (seconds)	133.00 (115.00, 160.50)	195.00 (158.00, 244.00)	< 0.001
MES scores	91.00 (85.00, 96.00)	76.00 (64.00, 85.00)	< 0.001
HAMD scores	2.00 (1.00, 5.00)	4.00 (2.00, 8.00)	< 0.001
HAMA scores	3.00 (1.00, 5.00)	5.00 (2.00, 9.00)	< 0.001
GDS scores	1.00 (1.00, 2.00)	2.00 (1.00, 5.00)	< 0.001
CDR scores	0.00 (0.00, 0.00)	0.50 (0.50, 0.50)	< 0.001
FAQ scores	0.00 (0.00, 0.00)	1.00 (0.00, 5.00)	< 0.001

Comprehensive neuropsychological assessment of the total study cohort (NC, n=96; aMCI, n=99). Data are presented as mean ± standard deviation (SD) for normally distributed continuous variables (analysed by Student's *t*-test) or as median with interquartile range (IQR) for non-normally distributed continuous variables (analysed by Mann-Whitney *U* test). $P < 0.05$ was considered statistically significant. Abbreviations: NC, normal controls; aMCI, amnestic mild cognitive impairment; MMSE, mini-mental state examination; MoCA-B, Montreal cognitive assessment-basic scores; BNT, Boston naming test; VFT, verbal fluency test; STT-A, shape trail test-part A; STT-B, shape trail test-part B; MES, memory and executive screening; HAMD, Hamilton depression rating scale; HAMA, Hamilton anxiety rating scale; GDS, global deterioration scale; CDR, clinical dementia rating; FAQ, functional activities questionnaire.

Supplementary Table 3 Demographical and clinical characteristics in *ApoE* ε4 non-carriers

**Variables**	**NC (n = 74)**	**aMCI (n = 64)**	***P***
Age, years	69.43 ± 8.37	70.16 ± 7.35	0.593
Female, n (%)	46 (62.16%)	37 (57.81%)	0.603
Education, years	13.50 (12.00, 16.00)	12.00 (9.00, 14.50)	< 0.001
MMSE scores	28.00 (27.00, 30.00)	26.00 (24.00, 28.00)	< 0.001
MoCA-B scores	26.00 (24.00, 27.25)	21.00 (18.25, 23.00)	< 0.001
BNT scores	26.00 (24.00, 28.00)	21.00 (18.00, 24.00)	< 0.001
VFT scores	19.76 ± 4.44	14.90 ± 4.92	< 0.001
STT-A (seconds)	53.00 (44.00, 65.00)	85.00 (60.00, 113.00)	< 0.001
STT-B (seconds)	133.00 (111.00, 160.00)	207.00 (163.00, 255.00)	< 0.001
MES scores	91.00 (85.00, 95.00)	76.00 (63.00, 85.00)	< 0.001
HAMD scores	2.00 (0.75, 4.25)	5.50 (2.00, 8.75)	< 0.001
HAMA scores	3.00 (1.00, 5.00)	6.00 (3.00, 10.00)	< 0.001
GDS scores	1.00 (1.00, 3.00)	3.00 (2.00, 5.00)	< 0.001
CDR scores	0.00 (0.00, 0.00)	0.50 (0.50, 0.50)	< 0.001
FAQ scores	0.00 (0.00, 0.00)	1.00 (0.00, 5.00)	< 0.001

Demographical and clinical characteristics in *ApoE* ε4 non-carriers (NC, n=74; aMCI, n=64). Data are presented as mean ± standard deviation (SD) for normally distributed continuous variables (analysed by Student's *t*-test) or as median with interquartile range (IQR) for non-normally distributed continuous variables (analysed by Mann-Whitney *U* test). Categorical variables are expressed as numbers (percentages) and were compared using the Chi-square test. $P < 0.05$ was considered statistically significant. Abbreviations: NC, normal controls; aMCI, amnestic mild cognitive impairment; MMSE, mini-mental state examination; MoCA-B, Montreal cognitive assessment-basic scores; BNT, Boston naming test; VFT, verbal fluency test; STT-A, shape trail test-part A; STT-B, shape trail test-part B; MES, memory and executive screening; HAMD, Hamilton depression rating scale; HAMA, Hamilton anxiety rating scale; GDS, global deterioration scale; CDR, clinical dementia rating; FAQ, functional activities questionnaire.

Supplementary Table 4 Demographical and clinical characteristics in *ApoE* ε4 carriers

**Variables**	**NC (n = 22)**	**aMCI (n = 35)**	***P***
Age, years	68.68 ± 6.64	70.51 ± 8.76	0.405
Female, n (%)	12 (54.55%)	24 (68.57%)	0.285
Education, years	15.00 (10.50, 16.00)	12.00 (9.00, 16.00)	0.179
MMSE scores	29.00 (27.50, 30.00)	24.50 (22.00, 27.00)	< 0.001
MoCA-B scores	25.00 (24.00, 27.00)	20.00 (19.00, 23.00)	< 0.001
BNT scores	26.00 (23.50, 28.00)	23.00 (20.00, 25.00)	< 0.001
VFT scores	18.00 (15.00, 22.25)	13.00 (11.00, 16.00)	< 0.001
STT-A (seconds)	54.00 (44.00, 62.00)	69.50 (54.25, 100.75)	0.004
STT-B (seconds)	133.00 (117.75, 170.00)	185.00 (144.25, 214.50)	< 0.001
MES scores	91.00 (87.50, 95.75)	75.00 (64.00, 83.00)	< 0.001
HAMD scores	2.00 (0.00, 5.00)	3.00 (1.00, 7.00)	0.272
HAMA scores	4.00 (1.50, 6.50)	5.00 (2.00, 7.00)	0.528
GDS scores	1.00 (1.00, 2.00)	2.00 (1.00, 4.00)	0.047
CDR scores	0.00 (0.00, 0.00)	0.50 (0.50, 0.50)	< 0.001
FAQ scores	0.00 (0.00, 0.00)	2.00 (0.00, 5.00)	0.002

Demographical and clinical characteristics in *ApoE* ε4 carriers (NC, n=22; aMCI, n=35). Data are presented as mean ± standard deviation (SD) for normally distributed continuous variables (analysed by Student's *t*-test) or as median with interquartile range (IQR) for non-normally distributed continuous variables (analysed by Mann-Whitney *U* test). Categorical variables are expressed as numbers (percentages) and were compared using the Chi-square test. $P < 0.05$ was considered statistically significant. Abbreviations: NC, normal controls; aMCI, amnestic mild cognitive impairment; MMSE, mini-mental state examination; MoCA-B, Montreal cognitive assessment-basic scores; BNT, Boston naming test; VFT, verbal fluency test; STT-A, shape trail test-part A; STT-B, shape trail test-part B; MES, memory and executive screening; HAMD, Hamilton depression rating scale; HAMA, Hamilton anxiety rating scale; GDS, global deterioration scale; CDR, clinical dementia rating; FAQ, functional activities questionnaire.

Supplementary Table 5 Correlations analysis of C4b and FB with neuropsychological scores in the total cohort

**Domain**	**Assessment Tool**	**C4b (*r, P*)**	**FB (*r, P*)**
**Global Cognition**	MMSE	-0.264, 0.001	-0.079, 0.329
	MoCA-B	-0.283, < 0.001	-0.121, 0.104
**Language**	BNT	-0.198, 0.008	0.021, 0.779
	VFT	-0.323, < 0.001	-0.125, 0.095
**Executive Function & Attention**	STT-A	0.294, < 0.001	0.078, 0.297
	STT-B	0.241, 0.001	0.008, 0.914
**Memory & Executive Screening**	MES	-0.287, < 0.001	-0.125, 0.096
**Mood**	HAMD	0.068, 0.368	-0.097, 0.195
	HAMA	0.121, 0.112	-0.077, 0.306
**Disease Severity & Staging**	GDS	0.178, 0.019	0.032, 0.675
	CDR	0.292, < 0.001	0.150, 0.071
**Daily Function**	FAQ	0.212, 0.006	0.085, 0.270

Correlations analysis of C4b and FB with neuropsychological scores in the total cohort (NC, n=96; aMCI, n=99). A correlation analysis was performed using Spearman's rank correlation coefficient, given the non-normal distribution of the data. Correlation coefficients (r) and corresponding *P*-values are presented for each pairwise comparison. $P < 0.05$ was considered statistically significant. Abbreviations: MMSE, mini-mental state examination; MoCA-B, Montreal cognitive assessment-basic scores; BNT, Boston naming test; VFT, verbal fluency test; STT-A, shape trail test-part A; STT-B, shape trail test-part B; MES, memory and executive screening; HAMD, Hamilton depression rating scale; HAMA, Hamilton anxiety rating scale; GDS, global deterioration scale; CDR, clinical dementia rating; FAQ, functional activities questionnaire.

Supplementary Table 6 Correlations analysis of C4b and FB with neuropsychological scores in *ApoE* ε4 non-carriers

Domain	Assessment Tool	C4b (*r*, *P*)	FB (*r*, *P*)
**Global Cognition**	MMSE	-0.216, 0.028	-0.041, 0.678
	MoCA-B	-0.242, 0.006	-0.097, 0.278
**Language**	BNT	-0.214, 0.015	0.034, 0.704
	VFT	-0.323, < 0.001	-0.083, 0.354
**Executive Function & Attention**	STT-A	0.240, 0.007	0.027, 0.760
	STT-B	0.196, 0.030	-0.016, 0.857
**Memory & Executive Screening**	MES	-0.214, 0.016	-0.123, 0.165
**Mood**	HAMD	0.084, 0.349	-0.043, 0.632
	HAMA	0.082, 0.364	-0.024, 0.792
**Disease Severity & Staging**	GDS	0.164, 0.069	0.020, 0.824
	CDR	0.225, 0.026	0.058, 0.567
**Daily Function**	FAQ	0.103, 0.258	-0.012, 0.892

Correlations analysis of C4b and FB with neuropsychological scores in *ApoE* ε4 non-carriers (NC, n=74; aMCI, n=64). A correlation analysis was performed using Spearman's rank correlation coefficient, given the non-normal distribution of the data. Correlation coefficients (r) and corresponding *P*-values are presented for each pairwise comparison. $P < 0.05$ was considered statistically significant. Abbreviations: MMSE, mini-mental state examination; MoCA-B, Montreal cognitive assessment-basic scores; BNT, Boston naming test; VFT, verbal fluency test; STT-A, shape trail test-part A; STT-B, shape trail test-part B; MES, memory and executive screening; HAMD, Hamilton depression rating scale; HAMA, Hamilton anxiety rating scale; GDS, global deterioration scale; CDR, clinical dementia rating; FAQ, functional activities questionnaire.

Supplementary Table 7 Correlations analysis of C4b and FB with neuropsychological scores in *ApoE* ε4 carriers

**Domain**	**Assessment Tool**	**C4b (*r*, *P*)**	**FB (*r*, *P*)**
**Global Cognition**	MMSE	-0.290, 0.048	-0.170, 0.237
	MoCA-B	-0.399, 0.004	-0.192, 0.173
**Language**	BNT	-0.152, 0.296	-0.051, 0.719
	VFT	-0.274, 0.056	-0.219, 0.119
**Executive Function & Attention**	STT-A	0.402, 0.004	0.249, 0.075
	STT-B	0.317, 0.026	0.053, 0.708
**Memory & Executive Screening**	MES	-0.464, 0.001	-0.162, 0.260
**Mood**	HAMD	0.031, 0.833	-0.213, 0.129
	HAMA	0.126, 0.221	-0.255, 0.068
**Disease Severity & Staging**	GDS	0.232, 0.109	0.062, 0.664
	CDR	0.451, 0.002	0.374, 0.010
**Daily Function**	FAQ	0.427, 0.004	0.316, 0.030

Correlations analysis of C4b and FB with neuropsychological scores in *ApoE* ε4 carriers (NC, n=22; aMCI, n=35). A correlation analysis was performed using Spearman's rank correlation coefficient, given the non-normal distribution of the data. Correlation coefficients (*r*) and corresponding *P*-values are presented for each pairwise comparison. $P < 0.05$ was considered statistically significant. Abbreviations: MMSE, mini-mental state examination; MoCA-B, Montreal cognitive assessment-basic scores; BNT, Boston naming test; VFT, verbal fluency test; STT-A, shape trail test-part A; STT-B, shape trail test-part B; MES, memory and executive screening; HAMD, Hamilton depression rating scale; HAMA, Hamilton anxiety rating scale; GDS, global deterioration scale; CDR, clinical dementia rating; FAQ, functional activities questionnaire.
