# Supplementary material for: Complement C4b and factor B in astrocyte-derived exosomes as novel biomarkers for amnestic mild cognitive impairment
Source: Front Aging Neurosci. 2026 Sep 30;18:1727449. doi: 10.3389/fnagi.2026.1727449 (PMC13661567; doi:10.3389/fnagi.2026.1727449)
Supplement: Supplementary file 2 [file Data_Sheet_2.pdf]

Supplementary Figure legends

Supplementary Figure 1 (A-D) Presented are the complete, uncropped Western blot images corresponding to Figure 1C in the main text, illustrating the expression profiles of four marker proteins across various fractions during the total exosome isolation process: (A) HSP70, serving as an exosome-positive marker; (B) CD63, another exosome-positive marker; (C) Calnexin, an indicator of endoplasmic reticulum contamination; and (D) Albumin, utilized as a control for high-abundance plasma proteins. The lanes are designated as follows: plasma (P), total exosomes (Exos), microvesicles (MVs), fibrin pellet (Fibrin), and exosomes -depleted plasma supernatant (EDP). The findings indicate that HSP70 and CD63 were predominantly enriched in the Exos fraction. Calnexin was exclusively detected in the MVs fraction, thereby confirming the absence of endoplasmic reticulum contamination in the exosome preparations. Albumin exhibited high expression levels in the P and EDP fractions but was scarcely detectable in the Exos fraction, suggesting that the exosomes precipitated using ExoQuick were not substantially contaminated by high-abundance plasma proteins. (E) The complete, uncropped Western blot image corresponding to Figure 1D in the main text illustrates the expression of the astrocyte-specific marker GFAP in immunoaffinity-enriched ADEs. Samples were collected from four representative NC subjects and four patients with aMCI. The presence of GFAP bands in all ADE samples indicates that the enriched ADEs predominantly originate from astrocytes. (F) Representative Western blot images of exosomal and cellular origin markers in ADE samples from three NC subjects and three aMCI patients are presented. The markers analyzed include Alix (a general exosomal marker), GFAP (an astrocyte marker), CD171 (a neuron-derived exosome marker), and CD11b (a microglia-derived exosome marker). The findings reveal that all ADE samples exhibited high expression levels of Alix and GFAP, while CD171 and CD11b were not expressed. This further corroborates that the immunoaffinity-enriched ADEs are primarily derived from astrocytes, rather than neurons or microglia.
Abbreviations: ADEs, astrocyte-derived exosomes; GFAP, glial fibrillary acidic protein; NC, normal control; aMCI, amnestic mild cognitive impairment.

Supplementary Figure 2 Unadjusted levels of C4b and FB in ADEs from the total cohort (NC, n=96; aMCI, n=99). (A) Comparison of C4b levels in ADEs between the NC and aMCI groups without adjustment for sex, age, years of education, and *ApoE* ε4 genotype. (B) Comparison of FB levels in ADEs between the NC and aMCI groups without adjustment for the above covariates. * $P < 0.05$; ** $P < 0.01$; *** $P < 0.001$; ns, not significant. Note: The analyses presented in this supplementary figure were performed without covariate adjustment. Adjusted results (accounting for sex, age, years of education, and *E* ε4 genotype) are shown in the main text (Figure 2A and 2B).
Abbreviations: ADEs, astrocyte-derived exosomes; NC, normal control; aMCI, amnestic mild cognitive impairment; *ApoE* ε4, Apolipoprotein E ε4.

Supplementary Figure 3 Unadjusted levels of C4b and FB in ADEs from *ApoE* ε4 non-carriers (NC, n=74; aMCI, n=64). (A) Comparison of C4b levels in ADEs between the NC and aMCI groups without adjustment for sex, age, and years of education. (B) Comparison of FB levels in ADEs between the NC and aMCI groups without adjustment for the above covariates. * $P < 0.05$; ** $P < 0.01$; *** $P < 0.001$; ns, not significant. Note: The analyses presented in this supplementary figure were performed without covariate adjustment. Adjusted results (accounting for sex, age, and years of education) are shown in the main text (Figure 3A and 3B).
Abbreviations: ADEs, astrocyte-derived exosomes; NC, normal control; aMCI, amnestic mild cognitive impairment; *ApoE* ε4, Apolipoprotein E ε4.

Supplementary Figure 4 ROC curve analysis of different logistic regression models for discriminating aMCI from NC in *ApoE* ε4 non-carriers (NC, n=74; aMCI, n=64). The baseline model incorporated age, sex, and education, while extended models were developed by adding C4b alone, FB alone, or both C4b and FB to the baseline model. The ROC curves and the AUC values are depicted for each model. DeLong's test was employed to compare the AUCs of the extended models with that of the baseline model, and no statistically significant differences were found ($P > 0.05$ for all comparisons). This figure presents supplementary analyses; the main text (Figure 3C) only shows the comparison between the baseline model and the baseline model plus C4b.
Abbreviations: ROC, receiver operating characteristic; AUC, area under the curve; NC, normal control; aMCI, amnestic mild cognitive impairment; ADEs, astrocyte-derived exosomes.

Supplementary Figure 5 Unadjusted levels of C4b and FB in ADEs from *ApoE* ε4 carriers (NC, n=22; aMCI, n=35). (A) Comparison of C4b levels in ADEs between the NC and aMCI groups without adjustment for sex, age, and years of education. (B) Comparison of FB levels in ADEs between the NC and aMCI groups without adjustment for the above covariates. * $P < 0.05$; ** $P < 0.01$; *** $P < 0.001$; ns, not significant. Note: The analyses presented in this supplementary figure were performed without covariate adjustment. Adjusted results (accounting for sex, age, and years of education) are shown in the main text (Figure 4A and 4B).
Abbreviations: ADEs, astrocyte-derived exosomes; NC, normal control; aMCI, amnestic mild cognitive impairment; *ApoE* ε4, Apolipoprotein E ε4.
